# Disseminated intravascular coagulation following malaria due to *Plasmodium vivax*: a thromboelastography-based study

**DOI:** 10.1186/1475-2875-12-336

**Published:** 2013-09-22

**Authors:** Sarita Mohapatra, Jyotish C Samantaray, Subramaniyan Arulselvi, Arnab Ghosh

**Affiliations:** 1 Department of Microbiology, Vardhaman and Mahavir Medical College and Safdarjung Hospital, 5th floor, New Delhi 110029, India; 2 Department of Microbiology, All India Institute of Medical Sciences, New Delhi, India; 3 Unit of Laboratory Medicine, JPNA Trauma Centre;, All India Institute of Medical Sciences, New Delhi, India

**Keywords:** *Plasmodium vivax*, Severe malaria, Disseminated intravascular coagulation, Thromboelastography

## Abstract

**Background:**

Disseminated intravascular coagulation (DIC) due to *Plasmodium vivax* is scarcely reported in comparison to *Plasmodium falciparum*. In complicated malaria, thrombocytopaenia and haemostatic alterations lead to increased activation of coagulation cascade and fibrinolytic system. Thromboelastography (TEG) is a haemostasis system which measures the viscoelastic strength of blood clot in the coagulation pathway. It detects the initial derangement in clotting cascade involving in platelet interaction and fibrinolysis. Hence, it can document the early changes in coagulation in vitro, and thereby guide the management. The current study was aimed at detection of DIC in patients with *P. vivax* malaria based on TEG.

**Methods:**

Ethylene diamine tetraacetic acid (EDTA) blood samples from acute febrile patients were tested by microscopy and immunochromatographic test for malaria. A total of 31 confirmed cases of vivax malaria were enrolled for this study. All the samples were tested by thromboelastography and conventional tests parameters for detection of any coagulation derangement.

**Results:**

Of 31, 17 (55%) were classified as complicated and 14/31 (45%) were uncomplicated. Among 23 cases with thrombocytopaenia, non-overt (early stage) DIC was detected in 18 cases by TEG and 17 cases by the conventional methods.

**Conclusion:**

It seems that the burden of DIC in vivax malaria is much higher than the world literature reported. TEG can be utilized as an important tool for early detection of DIC and guiding the management in malaria patients.

## Background

Among the different species of *Plasmodium*, *Plasmodium falciparum* and *Plasmodium vivax* account for majority of malaria infection worldwide. Complicated malaria is observed to be a consequence of *P. falciparum* infection mainly [1]. In contrast, *P. vivax* is found to be relatively benign. However, many recent reports from Africa, Southeast Asia and India highlighted its association in causation of complicated malaria [2-5]. The complications in vivax malaria were observed in the form of severe anaemia, thrombocytopaenia, pulmonary oedema, renal failure, metabolic imbalance, and cerebral malaria, etc. Thrombocytopaenia is found to be more commonly associated with vivax in comparison to falciparum malaria [6]. It is associated with the activation of coagulation pathway followed by disseminated intravascular coagulation (DIC) in severe falciparum malaria [7,8]. But, apart from few case reports, there is no strong evidence to prove the above hypothesis in *P. vivax* infection. DIC is diagnosed as a combination of clinical features such as haemorrhagic manifestations and laboratory tests (i e, prothrombin time (PT), activated partial thromboplastin time (aPTT), D-dimers, fibrinogen degradation product, etc.). However, absence of bleeding is often considered as negative for DIC and misdiagnosed many non-overt (early) cases of DIC. Apart from routine laboratory tests, thromboelastograpy (TEG) is a popular method for early and accurate detection of DIC in trauma, organ transplant, cardiac surgery, and obstetric procedures [9]. TEG measures the changes in the coagulation in vitro, i e, from the initial clotting cascade, platelet interaction, clot strength and lastly the fibrinolysis. Hence, it can correctly predict the early onset of DIC before the patient manifests and guide the therapy. In the present study, was aimed to detect early onset of DIC in vivax malaria cases by TEG. It was also tried to find out the correlation of development of DIC with the severity of the disease.

## Methods

A prospective study was carried out over a period of four months in which patients with diagnosis of malaria were enrolled after receiving clearance from the institute ethics committee (All India Institute of Medical Sciences, New Delhi). Blood sample from acute febrile patient with clinical diagnosis of malaria was tested by microscopy (Giemsa, Acridine Orange (AO), and quantitative buffy coat assay (QBC)) and immunochromatographic test(ICT) (Advantage Mal Card, J Mitra & Co Pvt Ltd, location? New Delhi, India). Infection of *P. vivax* was confirmed in 31 blood samples by the above methods. Speciation was characterized and parasitaemia was calculated in all the positive slides as per WHO guidelines. PCR could not be performed in these samples. However, considering the high sensitivity and specificity of QBC and ICT, the possibility of mixed infection with *P. falciparum* was ruled out. All patients were categorized as complicated or uncomplicated as per WHO criteria [9]. Patients with history of coagulation disorders, liver transplant, or cardiac surgery were excluded from the study.

### Processing of blood sample in TEG [10]

Three ml blood was collected in citrate (0.129 M trisodium citrate) vacutainer tubes from all patients positive for *P. vivax* and tested by TEG. TEG consists of a cup which holds the blood. Citrated blood was reconstituted by adding 20 μl of CaCl_2_ to 340 μl of blood in the cup for thromboelastographic test. The cup oscillates through an angle of 4˚45 with a pin suspended freely in the blood by torsion wire. The torque of the rotating cup is transmitted to the pin, thereby monitoring the motion. The clot formation, clot strength, lysis, and rate of fibrin-platelet bond formation affects the magnitude of pin motion. The signals are monitored and converted into graphical form by the computer. Thus TEG documents the initial fibrin formation, clot strengthening, and fibrin-platelet bonding, and clot lysis detecting hypocoagulation, normal coagulation, hypercoagulation, and fibrinolysis. Abnormal values were calculated in comparison to the normal values given by the standard reference and analysed for different coagulation dysfunction.

Calculation of coagulation index (CI) by TEG

$$\begin{array}{ll} \mathrm{CI}=0.3258R & -0.1886K+0.1224\mathrm{MA}+0.0759\alpha \\ & -7.7922 \end{array}$$

NormalvalueofCIrangesfrom−3.0to+3.0

$$\mathrm{CI}>+3=\mathrm{Hypercoagulable}\;\mathrm{condition}\;\left(\mathrm{early}/\mathrm{non}-\mathrm{overt}\;\mathrm{DIC}\right)$$

$$\mathrm{CI}<-3=\mathrm{Hypocoagulable}\;\mathrm{condition}\;\left(\mathrm{late}/\mathrm{overt}\;\mathrm{DIC}\right)$$

All samples were also analysed for the conventional coagulation tests such as prothrombin time (PT), activated partial thromboplastin time (aPTT), fibrinogen degradation product, D-dimer, etc. The International society for haemostasis and thrombosis (ISTH) scoring algorithm was used to determine overt and non-overt DIC conditions [11]. Fully automated haematology analyser Sysmex-XE 2100 was used for other haematological parameters, such as haemoglobin, total leukocyte count and platelet count.

## Results

In the present study, 31 febrile patiens were diagnosed positive for *P. vivax*. Among them, 17 (55%) were classified as complicated and 14 (45%) as uncomplicated (as per WHO criteria). The parasitaemia in complicated and uncomplicated groups ranged from 200/μl to 36,000/μl and 200/μl to 56,000/μl, respectively. In the complicated group, severe anaemia (10/31, 59%) was the commonest complication followed by acute renal failure (5/31, 29%). The other complications observed were delirium and altered sensorium (3/17 cases, 18%), pulmory oedema (3/17 cases, 18%), jaundice (2/17 cases, 12%). Nine out of 17 (53%) patients in the complicated group required intensive care unit for the management of complications. The number of cases in complicated and uncomplicated groups were found to be 13 (76%) and nine (64%), respectively, with alteration of TEG; in comparison to 10(59%) and 7 (50%), respectively, as detected by the conventional techniques. However, only one patient out of 31 complained of haemorrhagic manifestation and was clinically diagnosed to have DIC. None of the patient progressed to overt DIC during this period. A total 23/31 (74%) patients (including all the patients in the complicated group) showed thrombocytopaenia (platelet <1.5 lakh/dL). Eighteen out of 23 (78%) patients with thrombocytopaenia were detected to have non-overt (early) DIC by thromboelastography and 17 out 23 (74%) by the conventional test parameters (Table 1). No significant correlation was found between the incidence of DIC and degree of parasitaemia.

**Table 1:** Coagulation parameters by TEG and conventional coagulation tests in patients with thrombocytopaenia

**Sl. no.**	**Platelet count (≤1.5lakh)**	**Criteria**	**R (5.0-11.0)**	**K (1.5-4.5)**	**MA (53.0-70.0)**	**α angle (29.0-43.0)**	**PT in sec (13.7)**	**aPTT in sec (29.5)**	**d-Dimer (<0.5)**	**Fibrinogen (318)**
1.	60,000	Complicated	9.5	2.5	60.5	55.02	12.3	31	0.5-1	290
2.	80,000	Uncomplicated	8.2	3.3	53.8	49.35	17.5	30.8	2-4	427
3.	17,000	Complicated	6.8	1.7	78.5	68.99	12.8	31.5	1-2	432
4.	41,000	Uncomplicated	8.8	3.0	53.8	53.82	17.3	32.9	1-2	304
5.	10,000	Complicated	9.0	8.7	36.2	35.5	15.6	27	1-2	280
6.	20,000	Uncomplicated	5.8	3.2	55.7	56.64	17	17.5	<0.5	526
7.	40,000	Uncomplicated	7.0	2.3	53.8	59.5	12.9	33.2	4-8	670
8.	20,000	Complicated	6.0	3.3	61.9	55.02	16.4	43	1-2	276
9.	50,000	Uncomplicated	8.0	5.2	87.6	36.89	16.2	35.1	2-4	324
10.	14,000	Complicated	3.2	0.8	71.9	78.7	12.5	28.1	1-2	311

R: time to initial fibrin formation (S); K: time to clot formation (S); α angle (degree), rate of clot formation; MA: maximum amplitude (mm), absolute clot strength.

## Discussion

The present study highlights a surge of complicated malaria cases caused by *P. vivax*. In this study, more than 50% of vivax cases were categorized as complicated malaria and required hospitalization. The available literature was searched which revealed isolated case reports of DIC as a consequence of vivax malaria [12,13]. In this study, 23 patients had thrombocytopaenia and majority (>70%) were detected to have non-overt DIC by TEG as well as the conventional coagulation tests. Most of the literature described the role of platelet in the derangement of coagulation pathway in severe falciparum infection [14]. Activated platelet plays a major role in initiation, and propagation of coagulation cascade [11]. Alteration in the coagulation cascade leads to alteration in the haemostasis followed by a hypercoagulable condition (non-overt/early DIC) [14]. In uncomplicated malaria cases, hypercoagulable condition is mild and reverts back to normal after the timely and adequate anti-malarial therapy. However, in severe cases, this condition may proceed to hypocoagulable condition (overt/late DIC), which further leads to haemorrhagic manifestations. Most of the routine laboratory coagulation tests (i e, PT, APTT, TT) are requested after the haemorrhagic manifestations and might have missed many early stages of non-overt DIC (i e, without haemorrhagic manifestations). This is the first study detecting early stage of DIC (non-overt) with vivax malaria. Most of the patients with complicated malaria in this study showed thrombocytopaenia as well as developed non-overt DIC. But with timely diagnosis and appropriate anti-malarial therapy, all the patients reverted back to normal conditions without significant morbidity or mortality. This study highlights that firstly the incidence of DIC in vivax malaria is supposed to be much higher than the literature says, and secondly, platelet might have a role in the causation of DIC in vivax malaria.

## Conclusion

Recently, many reports published show an increasing rate of severity, with vivax malaria presenting with a variety of complications such as renal failure, pulmonary oedema, severe anaemia, thrombocytopaenia, etc. This is the first study to emphasize coagulation dysfunction in vivax malaria. Despite the small number, the majority of patients in this study with thrombocytopaenia showed a clear coagulation derangement in TEG as well as the conventional tests. Thrombocytopaenia can be taken as a valid marker of development of coagulation dysfunction, irrespective of the severity of the disease. Moreover, TEG is an important tool in the early diagnosis and monitoring of the development of DIC in these patients.

## Abbreviations

DIC: Disseminated intravascular coagulation; ICT: Immunochromatographic test; TEG: Thromboelastogram; PT: Prothrombin time; aPTT: Activated partial thromboplastin time; TT: Thromboplastine time; AO: Acridine orange; QBC: Quantitative buffy coat assay; CI: Coagulation index.

## Competing interests

The authors have declared that they have no competing interests.

## Authors’ contributions

SM participated in the conception, design, interpretation of data and drafting of manuscript. JCS reviewed critically the manuscript for final approval. SA participated in interpretation, and analysis of data, and drafting of manuscript. AG helped in acquisition, interpretation and analysis of data. All authors read and approved the final manuscript.
